# Hepatitis B Virus X Protein Up-Regulates AKR1C1 Expression Through Nuclear Factor-Y in Human Hepatocarcinoma Cells

**DOI:** 10.5812/hepatmon.8792

**Published:** 2013-05-27

**Authors:** Kai Li, Shijia Ding, Ke Chen, Dongdong Qin, Jialin Qu, Sen Wang, Yanrui Sheng, Chengcheng Zou, Limin Chen, Hua Tang

**Affiliations:** 1 Department of Molecular Biology on Infectious Disease, Second Affiliated Hospital, Chongqing Medical University, Chongqing, China; 2 Department of Clinical Laboratory Diagnostics, Chongqing Medical University, Chongqing, China; 3 Institute of Blood Transfusion, Chinese Academy of Medical Sciences and Peking Union Medical College, Chengdu, China; 4 Toronto General Research Institute, University of Toronto, Toronto, Canada

**Keywords:** Hepatitis B Virus X Protein, HepG2 Cells, 3 alpha-beta, 20 beta-hydroxysteroid dehydrogenase

## Abstract

**Background:**

The hepatitis B virus X (HBx) protein has long been recognized as an important transcriptional transactivator of several genes. Human aldo-keto reductase family 1, member C1 (AKR1C1), a member of the family of AKR1CS, is significantly increased in HBx-expressed cells.

**Objectives:**

This study aimed to investigate the possible mechanism of HBx in regulating AKR1C1 expression in HepG2.2.15 cells and the role of AKR1C1 for HBV-induced HCC.

**Materials and Methods:**

RT-PCR was performed to detect AKR1C1 expression on mRNA level in HepG2 and HepG2.2.15 cell. The promoter activity of AKR1C1 was assayed by transient transfection and Dual-luciferase reporter assay system. The AKR1C1 promoter sequence was screened using the TFSEARCH database and the ALIBABA 2.0 software. The potential transcription factors binding sites were identified using 5’ functional deletion analysis and site-directed mutagenesis.

**Results:**

In this study, we found that HBx promoted AKR1C1 expression in HepG2.2.15 cells. Knockdown of HBx inhibited AKR1C1 activation. The role of HBx expression in regulating the promoter activity of human AKR1C1 gene was analyzed. The 5’functional deletion analysis identified that the region between -128 and -88 was the minimal promoter region of HBx to activate AKR1C1 gene expression. Site-directed mutagenesis studies suggested that nuclear factor-Y (NF-Y) plays an important role in this HBx-induced AKR1C1 activation.

**Conclusions:**

In HepG2.2.1.5 cell, HBx can promote AKR1C1 promoter activity and thus activates the basal transcription of AKR1C1 gene. This process is mediated by the transcription factor NF-Y. This study explored the mechanism for the regulation of HBV on AKR1C1 expression and has provided a new understanding of HBV-induced HCC.

## 1. Background

The hepatitis B virus (HBV) infection plays an important role in the occurrence of hepatocellar carcinoma (HCC). Although the exact mechanisms remain unclear, many factors have been involved in this HBV-associated carcinogenesis. HBx, a HBV-encoded transcriptional activator, plays an important role in virus replication (1) and hepatocarcinogensis. Pervious studies have showen that HBV proteins can regulate many host gene expressions (2-4), but the exact mechanism remains to be determined. The HBx protein, a 17-kDa protein encoded by HBV genome, activates several genes associated with tumorigenesis (5). Two possible mechanisms for the host gene activation were proposed: HBx binds various transcription factors, such as NF-κB, AP-2 and CREB, and participates directly in the control of host gene transcription (6-8); HBx also regulates host gene transcription indirectly by acting on cellular signaling pathways (9). Human aldo-keto reductase family 1, member C1 (AKR1C1), one of four members of the aldo-keto reductase (AKR1C) family, is a NAD(P)H-dependent oxidoreductase that catalyzes progesterone to its inactive form, 20α-hydroxyprogesterone. Altered expression of AKR1C1 has been involved in many diseases, For example, a clear association between increased expression of AKR1C1 and the development of cisplatin-resistance in human ovarian carcinoma cells was observed (10). Previous studies have observed altered expression of AKR1C1 in a variety of human cancers. Human breast and ovarian carcinoma have reduced expression (11) while over expression of AKR1C1 was observed in patients with non-small cell lung cancer (NSCLC). In addition, patients with high AKR1C1 expression had significantly higher incidence of early tumor recurrence and distant organ metastasis (12). HBx can up-regulate the expression of AKR1C1 in the H7402-X cell line, a stable hepatocellular carcinoma cell line with HBV X gene integration (13). A report by Pallai et al. described that NF-Y regulates the basal transcription of AKR1C1 in HepG2 cells (14). A study by Selga et al. suggested that the transcription factor SP1 regulates the overexpression of AKRC1 in MTX sensitive HT29 human colon cancer cells (15). But transcription factors involved in regulating the transcription of AKR1C1 gene induced by HBX were not identified. In this study, we explored the underlying mechanisms for HBx-induced AKR1C1 expression in HepG2.2.15 cells. We demonstrated that HBV infection increased the expression of AKR1C1 by activation of its promoter. In HepG2 cells, HBx increased the mRNA levels of AKR1C1. When expression of HBx in HepG2.2.15 cells was repressed by siRNA, AKR1C1 could be significantly inhibited, indicating that HBx regulates AKR1C1 expression. Furthermore, our data showed that a NF-Y binding site in the AKR1C1 core promoter plays a critical role in this regulation. Taken together, our data indicate that HBx activates AKR1C1 dependent of NF-Y, which provides a possible mechanism for HBV-induced HCC, and suggests a novel therapeutic target for the treatment of HCC.

## 2. Objectives

This study aimed to investigate the possible mechanism of HBx in regulating AKR1C1 expression in HepG2.2.15 cells and the role of AKR1C1 for HBV-induced HCC.

## 3. Materials and Methods

HepG2 cells were purchased from ATCC (American Type Culture Collection, USA), Hep2.2.15 cells were purchased from the Shanghai Second Military Medical University and kept by our laboratory. The HBV expression plasmid pCH-9/3091 was constructed by Michael et al. (Heidelberg University, Germany) and was donated by Dr. Lan Lin (Southwest Hospital affiliation of the Third Military Medical University, China). pCMV-Sport6 plasmid was obtained from ATCC (American Type Culture Collection, USA). pCMV-Sport6-HBx, pCMV-Sport6-HBs, pCMV-Sport6-HBc, pCMV-Sport6-HBp were constructed by our laboratory (3, 4). HBx recombinant adenovirus and GFP control recombinant adenovirus were donated from Prof. He Tongchuan (The University of Chicago Medical Center, Chicago, Illinois, USA.). pGL3-Basic and pRL-TK were purchased from Invitrogen (USA).

### 3.1. Cell Culture

The human hepatocarcinoma cell lines, HepG2 and HepG2.2.15 were grown in Hyclone’s medium (MEM) supplemented with 10% fetal bovine serum (FBS), 100U/ml penicillin and 100μg/ml streptomycin, 5mmol/l glutamine at 37℃ in a humidified incubator with 5% CO_2_.

### 3.2. Total RNA Extraction, Reverse Transcription and Polymerase Chain Reaction

Total cellular RNA was extracted using the Trizol reagent (Invitrogen) from cells 48h after transfection. cDNA was synthesized using PrimeScript® RT reagent Kit (Takara, Dalian, China) according to the manufacturer’s protocol. The PCR was performed with an initial denaturation at 95℃ for 5 min, followed by 28 cycles of denaturation at 94℃ for 1 min, annealing at 58℃ for 1 min and extension at 72℃ for 1 min. The primers used are as follows: AKR1C1, sense: 5’-TGCTCTTATAGCCTGTGAGG-3’, antisense: 5’-AAGGATGACATTCCACCTGG-3’; HBx, sense: 5’-CCCGTCTGTGCCTTCTCATC-3’, antisense: 5’-CCCAACTCCTCCCAGTCTTT-3’; β-actin, sense: 5’-CCTTCTACAATGAGCTGCGT-3’, antisense: 5’-CCTGGATAGCAACGTACATG-3’. The amplicon size of AKR1C1, HBx and β-actin was 674 bp, 199 bp and 147 bp, respectively. The PCR products were analyzed in 1% TAE agarose gel followed by scanning and analysis using the GELDOC2000 system (Bio-Rad).

### 3.3. HBx Knock-Down by siRNA Transient Tranfection

Cells were seeded in 6-well plates one day before transfection at a density of 5×105/well. The sequences of HBx siRNA were as follows: HBx-siRNA1, 5’-GCACTTCGCTTCACCTCTG-3’; HBx-siRNA2, 5’-GCAATGTCAACGACCGACC-3’ (3, 16); pgenesil1.1-scramble sequence was used as a control siRNA plasmid. 4 μg of HBx siRNA was transfected using lipofectamine reagent according to manufacturer’s protocols (Invitrogen). RNAs were extracted 48h after transfection for RT-PCR analysis.

### 3.4. Construction of the AKR1C1 Promoter-Luciferase Plasmids and 5’deletion Analysis

To assess the activity of the AKR1C1 promoter induced by HBV, we constructed the pGL3-Basic-AKR1C1-P plasmid, which contained a 241 bp 5’promoter region of AKR1C1 (14, 15). In order to accurately identify the AKR1C1 core promoter region essential for its activity, longer fragment of AKR1C1 gene promoter was constructed in pGl3-basic vector (Promega) according to Pallai et al. (14), using a common reverse primer and different forward primers to amplify different lengths of promoter fragments from the HepG2 genomic DNA. Kpn I and Xho I sites were introduced in the forward and reverse primers. PCR amplified promoter fragments -641/+59, -349/+59, -180/+59, -152/+59, -128/+59, -88/+59 and -49/+59 were cloned in the Kpn I and Xho I sites of the pGL3-basic vector (14, 15). All the constructs were verified by restriction endonuclease digestion and sequencing.

### 3.5. Co-Transfection and Luciferase Assays

HepG2 cells and HepG2.2.15 cells were seeded in 24-well plates at 0.5 × 10^5^cells/well one day before transfection. HepG2 cells were co-transfected with 0.4 μg of various AKR1C1 promoter luciferase reporter constructs, 0.2 μg of pRL-TK reporter plasmid (Promega), and 0.2 μg of pCMV-Sport6 (blank vector as control) or pCMV-Sport6-HBx. HepG2.2.15 cells were co-transfected with 0.4 μg of various AKR1C1 promoter luciferase reporter constructs and 0.2 μg of pRL-TK reporter plasmid. Luciferase activity analysis was performed and normalized to the renilla luciferase using the luciferase assay kit (Dual-Luciferase Reporter Assay System, Promega) two days after transfection. In order to ensure the quality of the data, each transfection experiment was repeated at least three times.

### 3.6. Site-Directed Mutagenesis

Overlapping PCR and inverse PCR were used to generate various mutant clones. The pAKR1C1-349/+59 promoter was utilized to generate the pAKR1C1-349/+59d-128/-88 and the pAKR1C1-641/+59 promoter was utilized to generate the pAKR1C1-641/+59d-349/-180 construct. The pAKR1C1-180/+59 promoter was utilized to generate SP-1 binding site mutant clone (pAKR1C1-180/+59Mut1), NF-Y and C/EBP mutant clone (pAKR1C1-180/+59Mut2) and P40X and USF mutant clone (pAKR1C1-180/+59Mut3). The transcription factor binding site for SP-1 was mutated using primers 5’-TAAGACTGCCTCTGTTTTTCCTCTCACATGC-3’; 5’-GCATGTGAGAGGAAAAACAGAGGCAGTCTTA-3’. Similarly, the transcription factor binding site for NF-Y and C/EBP were mutated using the forward and reverse primers 5’-TCCTCTCACATGCCTTCAGTTAACCAGCAGACAGTG-3’; 5’-CACTGTCTGCTGGTTACTGAAGGCATGTGAGAGGA-3’. The transcription factor binding sites for P40X and USF were mutated using the forward and reverse primers: 5’-TGCCATTGGTTAACCTACAGACAGTGTGCTCAG-3’, 5’-CTGAGCACACTGTCTGTAGGTTAACCAATGGCA-3’ (mutated bases underlined). All the mutated clones were verified by sequencing. The promoter activity of the mutated clones was examined by transient transfection and luciferase assay.

### 3.7. Statistical Analysis

Dates were expressed as mean ± SD of three determinations. Statistical analysis was performed for calculating the significant differences in relative luciferase activity.

## 4. Results

### 4.1. HBV Up-Regulated AKR1C1 Expression 

**Figure 1.**
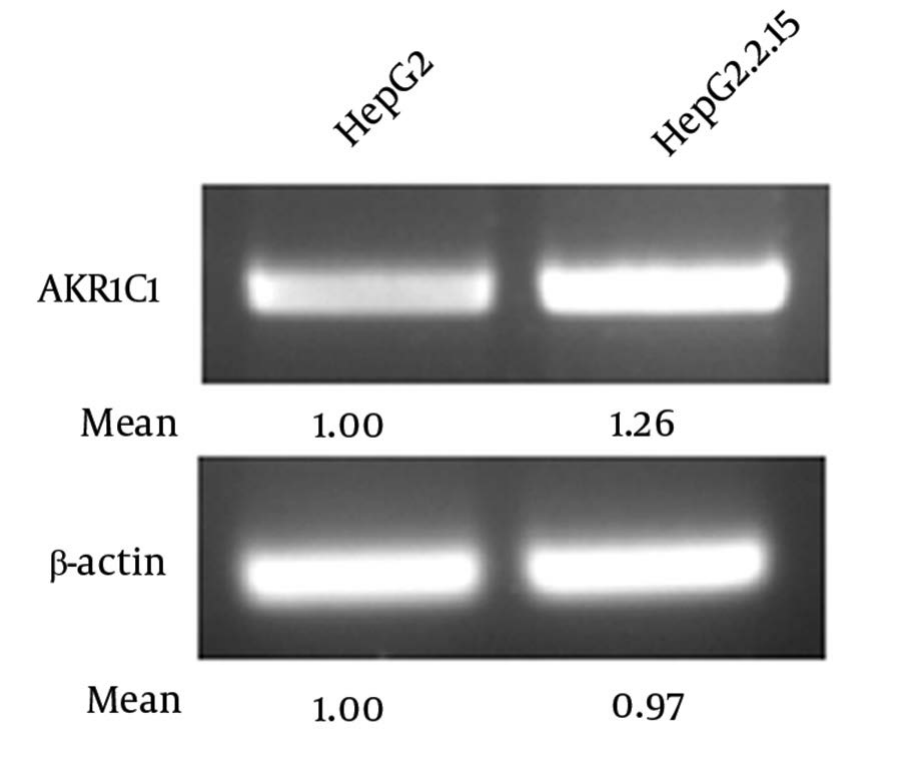
HBV Up-Regulated AKR1C1 Expression AKR1C1 expressions in HepG2 and HepG2.2.15 cells were analyzed with RT-PCR. β-actin was used as an internal quantitative control.

High-throughput gene expression profiling (microarray analysis) showed that AKR1C1 expression was increased in H7402-X cells stably transfected with hepatitis B virus X gene (13). In order to confirm this result, we compared AKR1C1 expression levels in HepG2 cells and HepG2.2.15 cells in which HBV proteins are stably expressed (17). Our data indicated that AKR1C1 mRNA level was significantly higher in HepG2.2.15 cells than that in HepG2 cells (Figure 1). Therefore, we concluded that the higher expression of AKR1C1 in HepG2.2.15 cells was due to HBV proteins in the cells.

### 4.2. HBV Up-Regulated AKR1C1 Expression by Promoting Its Promoter Activity and HBx Played an Important Role in This Process

**Figure 2.**
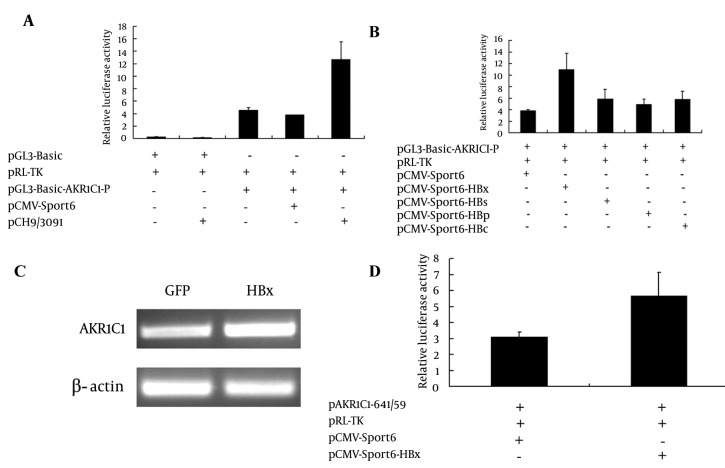
HBx Increased the Expression of AKR1C1 by Enhancing the Activity of Its Promoter A) 0.5 μg of pCH9/3091 was cotransfected with 0.5 μg of pGL3-Basic-AKR1C1-P and 0.2 μg of pRL-TK (Renilla expression vector) into HepG2 cells and luciferase assay was performed. Luciferase activity was normalized with the renilla luciferase activity in cell lysate. B) Four HBV expression plasmids (pCMV-Sport6-HBs, pCMV-Sport6-HBx, pCMV-Sport6-HBc, and pCMV-Sport6-HBp), pGL3-Basic-AKR1C1-P and pRL-TK were cotransfected into HepG2 cells, pCMV-Sport6 was used as the control and luciferase activity was measured. C) HBx recombinant adenovirus and GFP control recombinant adenovirus were used to infect the HepG2 cells and AKR1C1 expression were measured with RT-PCR. β-actin was used as a control. D) 0.2 μg of pAKR1C1-641/+59, 0.5 μg of pCMV-Sport6-HBx and 0.1 μg of pRL-TK were co-transfected into HepG2 cells. The pCMV-Sport6 group was used as a negative control. The values are means ± SD of three independent experiments.

To determine how HBV proteins regulated AKR1C1 gene expression, AKR1C1 gene promoter luciferase reporter plasmid (pGL3-Basic-AKR1C1-P) was generated. Data from co-transfection experiments showed that HBV increase AKR1C1 promoter activity 3.35 times compared to the control (Figure 2A, group 4 and group 5). HBV genome encodes four main proteins and in order to determine which protein has an influence on AKR1C1 promoter activity, each of the HBV protein expression plasmids (pCMV-Sport6-HBs, pCMV-Sport6-HBx, pCMV-Sport6-HBc, and pCMV-Sport6-HBp) were cotransfected with pGL3-Basic-AKR1C1-P and pRL-TK into HepG2 cells. As shown in Figure 2B, HBx could increase AKR1C1 promoter activity about 2.8 times, and the HBs, HBc and HBp protein had no significant effects on AKR1C1 promoter activity (Figure 2B). To further confirm that HBx could up-regulate the expression of AKR1C1, HBx expression adenovirus was used to infect the HepG2 cells and GFP (Green fFluorescencet pProtein) expression adenovirus was used as a control. As expected, HBx significantly increased AKR1C1 expression at the mRNA level (Figure 2C) and induced AKR1C1 promoter activity (Figure 2D). Taken together, these data demonstrated that HBx up-regulates AKR1C1 mRNA expression through activating its promoter activity.

### 4.3. Inhibition of HBx Decreased the Expression of AKR1C1 Through Suppression of the Promoter Activity 

**Figure 3.**
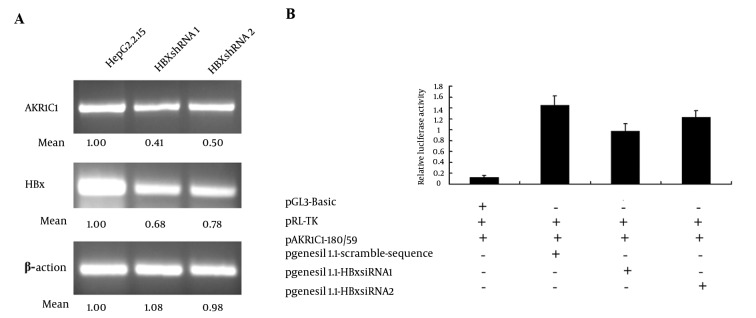
Inhibition of HBx Expression Decreased AKR1C1 Expression in HepG2.2.15 Cells A) HepG2.2.15 cells were transiently transfected with 4 μg of HBx siRNA. After 48 h, AKR1C1 and HBx expression were measured with RT-PCR and β-actin was used as an internal quantitative tool. B) HepG2.2.15 cells were cotransfected with 0.5 μg HBx siRNA, 0.2 μg of AKR1C1 promoter luciferase reporter construct (pAKR1C1-180/+59) and 0.1 μg of pRL-TK, pgenesil1.1-scramble sequence was used as a control siRNA plasmid, luciferase activity was measured by using the Dual-luciferase assay system. Luciferase activity was normalized with the renilla luciferase activity in cell lysate. Error bars indicate standard deviations (SD) obtained from the three different experiments prepared in triplicate. a *, P < 0.05; **, P < 0.01 as compared with the HBxsiRNA control groups

In order to further confirm the role of HBx in the process of up-regulation of the AKR1C1 expression, we attempted to knockdown the HBx expression by transient transfection of the HBx specific siRNA into HepG2.2.15 cells. As expected, when the HBx expression was inhibited by HBx siRNA, AKR1C1 expression was decreased (Figure 3A). Furthermore, when HBx siRNA, pAKR1C1-180/+59, pRL-TK were co-transfected into HepG2.2.15 cells, we found that HBx siRNA1 could significantly suppress the AKR1C1 gene promoter activity (Figure 3B). These results further demonstrated that HBx was a key regulator in AKR1C1 expression through affecting its promoter activity.

### 4.4. HBx Up-Regulated AKR1C1 Expression by Enhancing Its Promoter Activity Through -128/-88 Promoter Region

**Figure 4.**
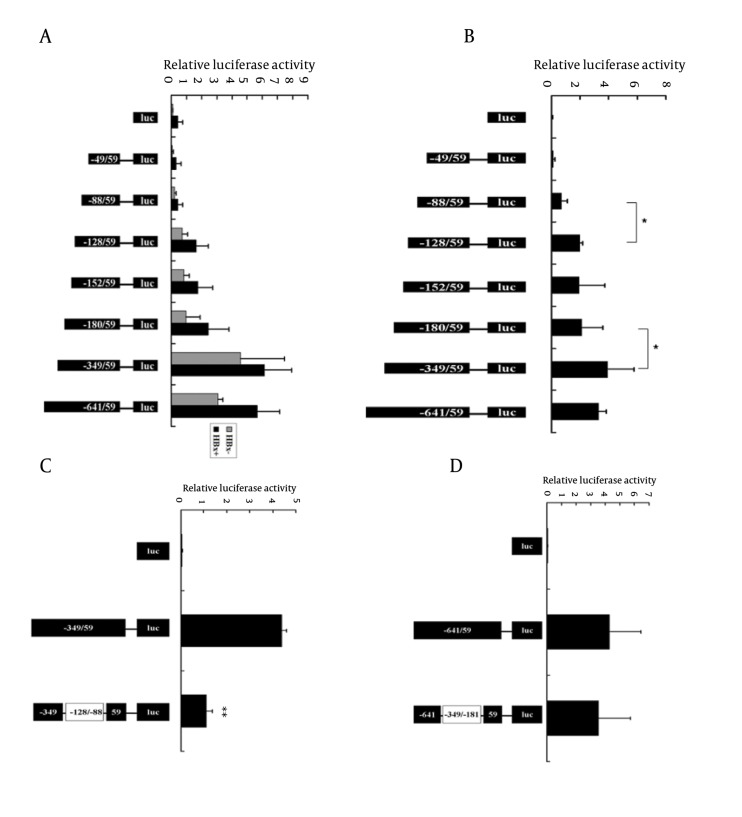
HBx Up-Regulated AKR1C1 Expression by Enhancing its Promoter Activity Through the -128/-88 Promoter Region A) 5’-Serial deletion construct of the promoter region of the AKR1C1 gene were co-transfected with pCMV-Sport6-HBx and pRL-TK in HepG2 cells, the relative luciferase activity was determined. HBx+ means these groups have been transfected with pCMV-Sport6-HBx, HBx- means these groups have been transfected with pCMV-Sport6 (control). B) The same constructs described as Fig.4A were transfected with pRL-TK in HepG2.2.15 cells and the relative luciferase activity was measured. C) HepG2.2.15 cells were transfected with 0.6 μg of pAKR1C1-349/+59 or pAKR1C1-349/+59d-128/-88 and 0.2 μg pRL-TK, and relative luciferase activities were measured. D) HepG2.2.15 cells were transfected with 0.6 μg of pAKR1C1-641/+59 or pAKR1C1-641/+59d-349/-181 and 0.2 μg pRL-TK, and luciferase assay were performed. On the left side of graph is the schematic representation of the AKR1C1 reporter gene constructs, and on the right side, the bar graphs represent the relative levels of luciferase activity. Error bars indicate standard deviations (SD) obtained from three different experiments prepared in triplicate. *, P < 0.05; **, P < 0.01 as compared with control groups

To determine which promoter region plays a critical role in the increased expression of AKR1C1 induced by HBx, luciferase reporter constructs containing several 5’-deletion of the AKR1C1 promoter sequence were co-transfected with pCMV-sport6-HBx into HepG2 cells. In the HBx transfection group, we found that the DNA fragments -49/+59 and-88/+59 show no activity above the pGL3-basic vector (Figure 4A). The activity of the construct containing the -349/+59 region showed the highest increase in relative luciferase activity (Figure 4A). The -128/+59 region displayed almost a 4-fold increase in luciferase activity compared to the -88/+59 region (Figure 4A). The activity of the pAKR1C1-349/+59 region was reduced by 2.5-folds, compared to the activity of the pAKR1C1-180/+59 (Figure 4A). In HepG2.2.15 cells, the pAKR1C1-49/+59 and pAKR1C1-88/+59 showed no activity compared to the negative control (Figure 4B, lane1-3). A 2.7 fold increase in relative luciferase activity was observed between the pAKR1C1-128/+59 and the pAKR1C1-88/+59. The activity of the construct containing -349/+59 was 1.84 fold higher than the pAKR1C1-180/+59. These data suggested that the region -128/-88 or the region -349/-180 might becritical for HBx-meidated activation. In order to determine which region had promoter activity induced by HBx, the mutant constructs (pAKR1C1-349/+59d-128/-88 and pAKR1C1-641/+59d-349/-180) were transfected with pRL-TK into HepG2.2.15 cells. As expected, the pAKR1C1-349/+59d-128/-88 displayed a 4-fold reduced activity compared to the pAKR1C1-349/+59 construct (Figure 4C, lane 3). In contrast, the pAKR1C1-641/+59d-349/-180 did not reduce the luciferase activity of the pAKR1C1-641/+59 construct (Figure 4D, lane 3). These results showed that HBx activated the AKR1C1 promoter through the critical promoter region between -128 and -88.

### 4.5. HBx activated the AKR1C1 expression in a NF-Y-dependent manner

**Figure 5.**
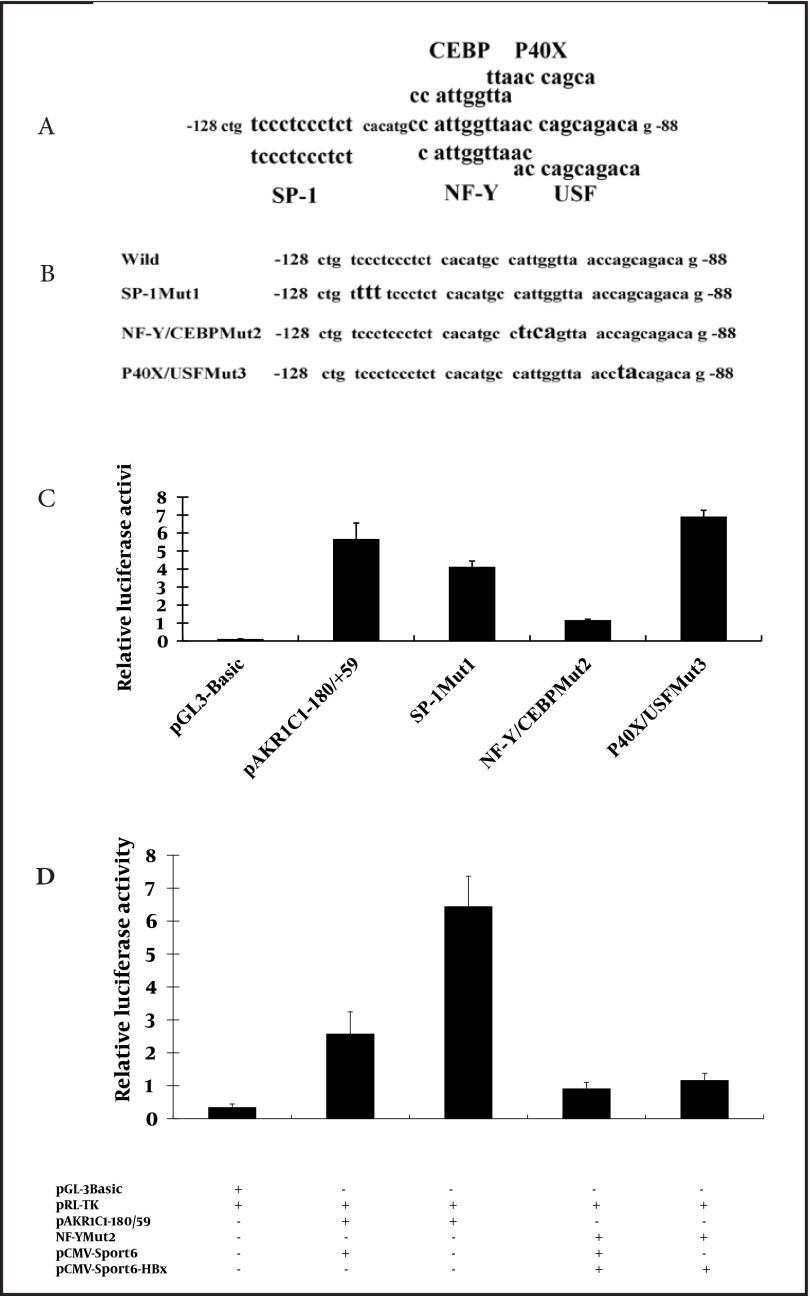
HBx Activated the AKR1C1 Expression in a NF-Y-dependent Manner A) The Alibaba 2.0 software and the TFSEARCH database was used to analyze the AKR1C1 promoter region between -128 and -88 and the potential transcription factor’s binding sites were identified. B) Three different mutants were obtained as described in materials and methods. Mut1 targeted the binding sites for the transcription factor SP1; Mut2 targeted the binding sites for the transcription factor NF-Y and CEBP; Mut3 targeted the p40X and USF binding site. C) HepG2.2.15 cells were transfected with pAKR1C1-180/+59Mut1, pAKR1C1-180/+59Mut2, pAKR1C1-180/+59Mut3, or the wild-type promoter (pAKR1C1-180/+59) and assayed for luciferase activity after 48 h. Transfection efficiency was normalized by co-transfection with pRL-TK. D) pAKR1C1-180/+59, NF-YMut2, and pCMV-sport6-HBx were co-transfected into HepG2 cells, pCMV-sport6 was used as the control and luciferase assay was performed. Transfection efficiency was normalized by co-transfection with pRL-TK. The mean ± SD are from three different experiments, each experiment performed in triplicate. *, P < 0.05; **P < 0.01 as compared with pCMV-Sport6 groups

The TFSEARCH database and the ALIBABA 2.0 software were used to screen for the possible binding factor in the -128/-88 region of the AKR1C1 promoter. Several transcription factors including SP-1, NF-Y, C/EBP, P40X, and USF were identified (Figure 5A). In order to demonstrate the involvement of these transcription factors in the HBx-mediated transactivation of AKR1C1 promoter, pAKR1C1-180/+59Muts (Figure 5B, pane 2-4) were co-transfected with pRL-TK in HepG2.2.15 cells. Dual-Luciferase activity assay revealed that the activity of the SP-1Mut2 was reduced by about 20% compared to the activity of the wild-type pAKR1C1-180/+59 construct (Figure 5C, lane 3). When pCMV-Sport6-SP-1 (a construct that expressed transcriptional factor SP-1), pAKR1C1-180/+59 and pRL-TK were co-transfected in HepG2.2.15 cells, the luciferase activity was not increased (data not shown).

Mutation in the NF-Y and C/EBP binding sites (pAKR1C1-180/+59Mut2) reduced luciferase activity by 4-folds compared to the wild-type pAKR1C1-180/+59 construct (Figure 5C, lane 4).

On the contrary, the activity of the P40X/USFMut3 is a little higher than the activity of the pAKR1C1-180/+59 (Figure 5C, lane 5). To confirm the role of HBx in the up-regulation of AKR1C1 gene in HepG2 cells, pAKR1C1-180/+59 or NF-YMut2,

pRL-TK and pCMV-sport6-HBx were co-transfected into HepG2 cells, with pCMV-sport6 as a negative control, and luciferase activity were measured. We found that HBx could significantly increase the activity of pAKR1C1-180/+59 about 2.5 times (Figure 5D, lane2-3). But the luciferase activity of NF-YMut2 was similar between the HBx transfected group and the control group (Figure 5D, lane 4-5). These results demonstrated that the C/EBP binding site, NF-Y, but not SP-1, P40X or USF, were involved in the induction of AKR1C1 by HBx in HepG2.2.15 cells.

## 5. Discussion

Chronic HBV infection is one of the risk factors associated with HCC (9, 18, 19). HBV-encoding proteins, such as HBx protein, play an important role in the development of HCC (5). HBx also plays a key role in HBV transcription and replication (1). Many studies have suggested that HBx functions through interaction with various transfactor’s, such as NF-κB, AP-2 and CREB, binding sites (6-8). AKR1C1 was one of the genes whose expression levels were altered in HBx transgenic cells identified by cDNA microarray analysis. AKR1C1 is a member of the aldo-keto reductase (AKR) supergene family, belonging to evolutionarily conserved NAD(P)H-dependent oxidoreductase class. Increased expression of AKR1C1 gene was observed in various cancer cells including lung (12), liver (14, 15) and ovarian (20). AKR1C1 is also involved in the development of drug resistance in various tumors (10, 14, 15, 21, 22). Although HBx-induced activation of AKR1C1 gene has been reported previously, the exact mechanism remains unclear. In this study, we aim to delineate the interaction between HBx and AKR1C1 to better understand how HBx activated AKR1C1. We found that HBx activated AKR1C1 gene expression through affecting its promoter activity, and the critical promoter region essential for this activation was identified. Furthermore, data from our study indicated that NF-Y was involved in this HBx-induced AKR1C1 activation. Our data indicate that HBV could up-regulate the expression of AKR1C1 (Figure 1A), and a similar result was observed between normal liver tissues and HBV-positive HCC cell lines (20). HBx has long been considered as the most important determinant in the virus-mediated pathogensis (5). In our study, we demonstrated that HBx induces the AKR1C1 expression through activating its promoter activity (Figure 2A, B, C and D). Suppression of the HBx expression in HepG2.2.15 cells by siRNA significantly reduces the AKR1C1 expression through suppressing its promoter activity (Figure 3A and B). To investigate the molecular mechanism of increased AKR1C1 expression induced by HBx, a series of deletion mutations of the 5’flanking region of the AKR1C1 gene promoter were created. Our results showed that the promoter region between -128 and -88 was critical for the transcriptional activation of AKR1C1 promoter by the HBx protein in HepG2.2.15 cells (Figure 4A, B, C and D). Pallai et al. (2010) identified that the transcription factor NF-Y binds to the inverted CCAAT box of the AKR1C1 using chromatin immunoprecipitation (CHIP) analysis and electrophoretic mobility shift (EMSA) assay. Their results showed that NF-Y regulated the basal transcription of AKR1C1 in HepG2 cells. Our data showed that -128/-88 promoter region is not only the minimal proximal promoter region (14, 15), but also the critical section for AKR1C1 expression regulated by HBx. NF-Y is a CCAAT-specific binding site, well-conserved transcriptional factor composed of three distinct subunits: NF-YA, NF-YB and NF-YC, all required for DNA-binding (23). It has been observed that the CCAAT box is a single element in the forward or reverse orientation between -60 and -100 of the major start site (24). Several promoters regulated by NF-Y have been identified, and all these promoters contain a CCAAT sequence close to the transcriptional start site. The CCAAT-box was essential for the basal expression of human phospholipid hydroperoxide glutathione peroxidase (PHGPx) gene in epidermoid carcinoma A431 cells (25), and the basal transcription of osteoclast differentiation factor (ODF) gene (26). NF-Y binding to CCAAT is considered to provide specificity in the mammalian unfolded protein response (UPR) (27). A previous study has indicated that NF-Y was identified in the basal regulation of the AKR1C1 gene in HepG2 cells (14), but the increased expression of AKR1C1 gene in HCC cells was not investigated. In our study, sequential deletion of a proximal AKR1C1 promoter assay was performed. Our results showed that the promoter region between -128 and -88 was critical for the up-regulation of AKR1C1 in HepG2.2.15 cells (Figure 4C). Analysis of AKR1C1 promoter by the TRANSFAC database and the ALIBABA 2.0 software revealed that transcription factor binding sites SP-1, NF-Y, C/EBP, P40X, and USF are in this region (Figure 5A). Site-directed mutagenesis demonstrated that NF-Y was responsible for the transcriptional activation of AKR1C1 gene in HepG2.2.15 cells (Figure 5C). HBx increased the activity of pAKR1C1-180/+59 significantly, but the activity of NF-Ymut2 (the mutate construct of NF-Y transcription factor binding site) was similar between the HBx group and the control group in HepG2 cells (Figure 5D). These data clearly demonstrated that HBx protein increased the basal transcription of AKR1C1 gene, and the CCAAT-box binding protein, NF-Y plays an important role in this regulation. Our data support previous studies demonstrating that HBx stimulate the AKR1C1 gene transcription at the promoter by enhancing the binding or activity of transcription factors (28, 29). Kabe’s report described that NF-Y is essential for the recruitment of RNAPⅡ onto the CCAAT box-containing promoters. Thus, the role of NF-Y in the formation of the transcription initiation complex warrants further studies. Some reports suggested that the c-Myc might interact with NF-Y (30) to regulate the cell cycle-dependent expression of the hsp70 gene (31). In line with this, interaction between NF-Y and other genes in HepG2.2.15 cells need further clarification. In conclusion, our results suggest that HBx activates the basal transcription of AKR1C1 gene by promoting its promoter activity in HepG2.2.15 cells and NF-Y play a critical role in this process. The involvement of HBx in up-regulation of AKR1C1 gene provides a new understanding and therapeutic target for HBV-induced HCC.
